# Draft Genome Sequence of Purine-Degrading *Gottschalkia purinilyticum* (Formerly *Clostridium purinilyticum*) WA1 (DSM 1384)

**DOI:** 10.1128/genomeA.01088-15

**Published:** 2015-09-24

**Authors:** Anja Poehlein, Frank R. Bengelsdorf, Bettina Schiel-Bengelsdorf, Rolf Daniel, Peter Dürre

**Affiliations:** a Genomic and Applied Microbiology & Göttingen Genomics Laboratory, Georg-August University, Göttingen, Germany; b Institut für Mikrobiologie und Biotechnologie, Universität Ulm, Ulm, Germany

## Abstract

Here, we report the draft genome sequence of *Gottschalkia purinilyticum* (formerly *Clostridium purinilyticum*) WA1, an anaerobic bacterium specialized on degradation of purines (including adenine) and glycine, which uses the selenoprotein glycine reductase for substrate degradation. The genome consists of a single chromosome (3.40 Mb).

## GENOME ANNOUNCEMENT

Few anaerobic bacteria are known that are specialized on purine degradation. These are *Gottschalkia acidurici* (formerly *Clostridium acidurici*) (1–3), *Clostridium cylindrosporum* (1, 2, 4), and *Gottschalkia purinilyticum* (formerly *Clostridium purinolyticum* and *C. purinilyticum*) (5). *G. purinilyticum* shows a strict selenium dependency (6) and could only be isolated by adding selenite to the enrichment medium (5). It differs from the other two species by its ability to grow on adenine. The pathway used for selenium-dependent purine degradation is identical to that determined for *G. acidurici* and *C. cylindrosporum* (7, 8). If no selenium is present, uric acid instead of xanthine is the central intermediate for degradation and is decomposed via pyrimidine derivatives (9). The genome sequences of *G. acidurici* (10) and *C. cylindrosporum* (11) have already been reported, so this report closes the gap for the specialized purine-degrading anaerobes.

The MasterPure complete DNA purification kit (Epicentre, Madison, WI, USA) was used to isolate chromosomal DNA. Illumina shotgun sequencing libraries were generated from the extracted DNA, and sequencing was performed with a Genome Analyzer IIx, as recommended by the manufacturer (Illumina, San Diego, CA, USA). Sequencing resulted in 13,923,714 reads that were trimmed using Trimmomatic version 0.32 (12). Five million of these reads were used for *de novo* assembly performed with the SPAdes genome assembler software version 3.5.0 (13). The assembly resulted in 74 contigs (>500 bp) and an average coverage of 165-fold. The genome of *G. purinilyticum* probably consists of a circular chromosome (3.40 Mb) with an overall G+C content of 28.83%.

Automatic gene prediction was performed by using the software tool Prodigal (14). Genes coding for rRNA and tRNA were identified using RNAmmer (15) and tRNAscan (16), respectively. The Integrated Microbial Genomes–Expert Review (IMG-ER) system (17) was used for automatic annotation, which was subsequently manually curated by using the Swiss-Prot, TrEMBL, and InterPro databases (18).

The genome harbored 5 rRNA genes, 47 tRNA genes, 2,430 protein-coding genes with predicted functions, and 856 genes coding for hypothetical proteins.

Genes coding for enzymes involved in the methyl branch of the Wood-Ljungdahl pathway were found, which explains the ability of *G. purinilyticum* to form acetate from CO_2_ via glycine (19). Genome analysis also revealed the presence of genes encoding a glycine reductase, which had been identified earlier by labeling experiments (6) and enzymatic determination (8). In contrast to *C. cylindrosporum*, the genome of *G. purinilyticum* harbors a gene cluster coding for a betaine reductase. The organization of this cluster seems to be highly conserved as we found identical clusters in *Eubacterium acidaminophilum*, *C. litorale*, and *C. sporogenes* (20–22). We could also identify genes coding for an electron-bifurcating formate dehydrogenase as recently described for *G. acidurici* (23). These genes are part of a large cluster consisting of *hylCBA-fdhF2-rbr2-fdhF1-rbr1-mogA-mobA-fdhD*, showing an arrangement similar to that of *G. acidurici* (10, 23).

### Nucleotide sequence accession numbers.

This whole-genome shotgun project has been deposited at DDBJ/EMBL/GenBank under the accession number LGSS00000000. The version described in this paper is version LGSS01000000.
